# The representation of shape and texture in category‐selective regions of ventral‐temporal cortex

**DOI:** 10.1111/ejn.15737

**Published:** 2022-06-21

**Authors:** David D. Coggan, David M. Watson, Ao Wang, Robert Brownbridge, Christopher Ellis, Kathryn Jones, Charlotte Kilroy, Timothy J. Andrews

**Affiliations:** ^1^ Department of Psychology University of York York UK; ^2^ Department of Psychology Vanderbilt University Nashville Tennessee USA

**Keywords:** adaptation, faces, FFA, fMRI, MVPA, places, PPA

## Abstract

Neuroimaging studies using univariate and multivariate approaches have shown that the fusiform face area (FFA) and parahippocampal place area (PPA) respond selectively to images of faces and places. The aim of this study was to determine the extent to which this selectivity to faces or places is based on the shape or texture properties of the images. Faces and houses were filtered to manipulate their texture properties, while preserving the shape properties (spatial envelope) of the images. In Experiment 1, multivariate pattern analysis (MVPA) showed that patterns of fMRI response to faces and houses in FFA and PPA were predicted by the shape properties, but not by the texture properties of the image. In Experiment 2, a univariate analysis (fMR‐adaptation) showed that responses in the FFA and PPA were sensitive to changes in both the shape and texture properties of the image. These findings can be explained by the spatial scale of the representation of images in the FFA and PPA. At a coarser scale (revealed by MVPA), the neural selectivity to faces and houses is sensitive to variation in the shape properties of the image. However, at a finer scale (revealed by fMR‐adaptation), the neural selectivity is sensitive to the texture properties of the image. By combining these neuroimaging paradigms, our results provide insights into the spatial scale of the neural representation of faces and places in the ventral‐temporal cortex.

## Graphical abstract

The aim of this study was to investigate the spatial scale at which shape and texture properties of objects are represented in high‐level visual cortex. We presented images of faces and places while recording fMRI in face‐ and scene‐selective regions of the brain. Coarser‐scale responses, measured through multivariate analyses showed sensitivity to shape but not texture properties. Finer‐scale responses, measured through fMR‐adaptation, showed sensitivity to both shape and textural properties.

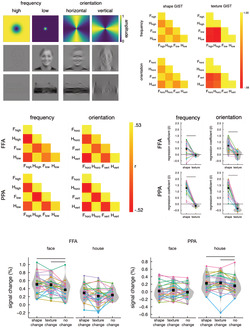

**Abbreviations**
ANOVA: analysis of variance
EPI: echo planar imaging
FFA: fusiform face area
fMR(I): functional magnetic resonance (imaging)
FOV: field of view
FWHM: full width at half maximum
HRF: haemodynamic response function
MVPA: multivariate pattern analysis
OFA: occipital face area
OPA: occipital place area
PPA: parahippocampal place area
pSTS: posterior superior temporal sulcus
ROI: region of interest
RSC: retrosplenial complex
RSM: representational similarity matrix
SD: standard deviation
SEM: standard error of the mean
TE: echo time
TR: repetition time
YNiC: York Neuroimaging Centre

## 1 INTRODUCTION

There is an important distinction between shape and texture properties in visual object perception. Many studies have shown the importance of the shape or spatial envelope of an object in recognition (Biederman, 1987; Grill‐Spector et al., 1998; Malach et al., 1995; Op de Beeck et al., 2008). However, other studies have shown that texture properties also provide important information for the perception and recognition of objects and faces (Andrews et al., 2016; Cant et al., 2009; Cant & Goodale, 2007, 2011; Cavina‐Pratesi et al., 2010; Lowe et al., 2016, 2017; Park & Park, 2017). Neuroimaging methods, such as fMRI, are able to discriminate object category in high‐level visual cortex. However, it is not always clear if the selectivity revealed by these methods is based primarily on the shape or texture properties of the images.

The most common univariate method involves cognitive subtraction, in which the response to an experimental condition is compared with a control condition in each voxel (Friston et al., 1996; Petersen et al., 1988). This has been used to reveal discrete regions in the ventral temporal lobe that are specialized for different categories of objects. For example, the fusiform face area (FFA) shows greater neural response to images of faces than to nonface objects, such as scenes or buildings (Kanwisher et al., 1997; McCarthy et al., 1997). In contrast, the parahippocampal place area (PPA) is more responsive to images of scenes and buildings compared with faces (Epstein & Kanwisher, 1998). However, a potential limitation of cognitive subtraction is that it may not be sensitive to different subpopulations of neurons within a voxel (Andrews, 2005; Avidan et al., 2002). This problem can be overcome with fMR‐adaptation or repetition suppression paradigms, in which the repetition of a stimulus causes a reduction or habituation in the neural response and a lower fMRI signal (Grill‐Spector et al., 1999; Grill‐Spector & Malach, 2001). A number of studies have shown selective adaptation to faces in the FFA (Andrews & Ewbank, 2004; Avidan et al., 2002; Coggan et al., 2019; Rotshtein et al., 2005) and houses in the PPA (Andrews et al., 2010; Avidan et al., 2002; Coggan et al., 2019; Epstein et al., 2003; Ewbank et al., 2005). The sensitivity of these neural representations can then be further investigated by changing the stimulus. If the underlying neural representation is sensitive to this change, the fMRI signal will increase towards the unadapted level (release from adaptation).

Multivariate pattern analysis (MVPA) in fMRI measures the pattern of response across many voxels (Haxby et al., 2014; Tong & Pratte, 2012). Using MVPA, distinct patterns of response have been reported for different categories of objects, including faces and houses (Haxby et al., 2001). Multivariate approaches are able to discriminate a greater range of object categories compared with univariate approaches (Haxby et al., 2001; Kriegeskorte et al., 2008). For example, the pattern of response in the PPA has been shown to differentiate between different types of scenes (Walther et al., 2009; Watson et al., 2014, 2017). Because the ability to differentiate between object categories is still evident after spatial smoothing, it is typically assumed that MVPA reflects a coarser‐scale representation (Op de Beeck et al., 2010; Rice et al., 2014; Watson et al., 2014).

The aim of this study is to combine univariate and multivariate analyses to explore the selectivity in the FFA and PPA to faces and houses. The rationale for using these different paradigms is that they provide sensitivity to coarser‐scale (MVPA) and finer‐scale (fMR‐adaptation) information about the neural response (Drucker & Aguirre, 2009; Epstein & Morgan, 2012; Hatfield et al., 2016; O'Connell & Chun, 2018). In previous studies, we have shown that different categories of objects vary in both shape and texture (Rice et al., 2014; Watson et al., 2014; Watson, Hymers, et al., 2016). Here, we attempt to determine the relative importance of shape and texture in the neural response by filtering the images (by orientation or frequency) to change their texture, but not their shape. In Experiment 1, a multivariate analysis compared the patterns of response to the filtered images in the FFA and PPA. If the pattern of response is dependent on shape, then applying a filter to the images should not change the patterns of response to faces and houses. In contrast, if the pattern of response is dependent on the texture, then applying a filter should change the patterns of response. In Experiment 2, an fMR‐adaptation paradigm (univariate) was used to determine the sensitivity to changes in texture properties caused by filtering. If there is a release from adaptation for a change in filter, this would suggest that the underlying representation is sensitive to the texture of the image.

## 2 METHODS

### 2.1 Participants

Twenty participants took part in Experiment 1 (eight male, median/min/max age = 25/18/35, SD = 4.8 years) and 24 participants took part in Experiment 2 (13 male, median/min/max age = 23/21/54, SD = 8.9 years). Sample size was arbitrarily based on previous studies using similar designs in which significant effects were evident (Coggan, Liu, et al., 2016; Watson et al., 2017). All participants were right‐handed, had normal or corrected‐to‐normal vision, and were neurologically healthy. Each gave their informed, written consent and the studies were approved by the York Neuroimaging Centre (YNiC) Ethics Committee and adhered to the original wording (1964) of the Declaration of Helsinki.

### 2.2 Stimuli

**FIGURE 1:**
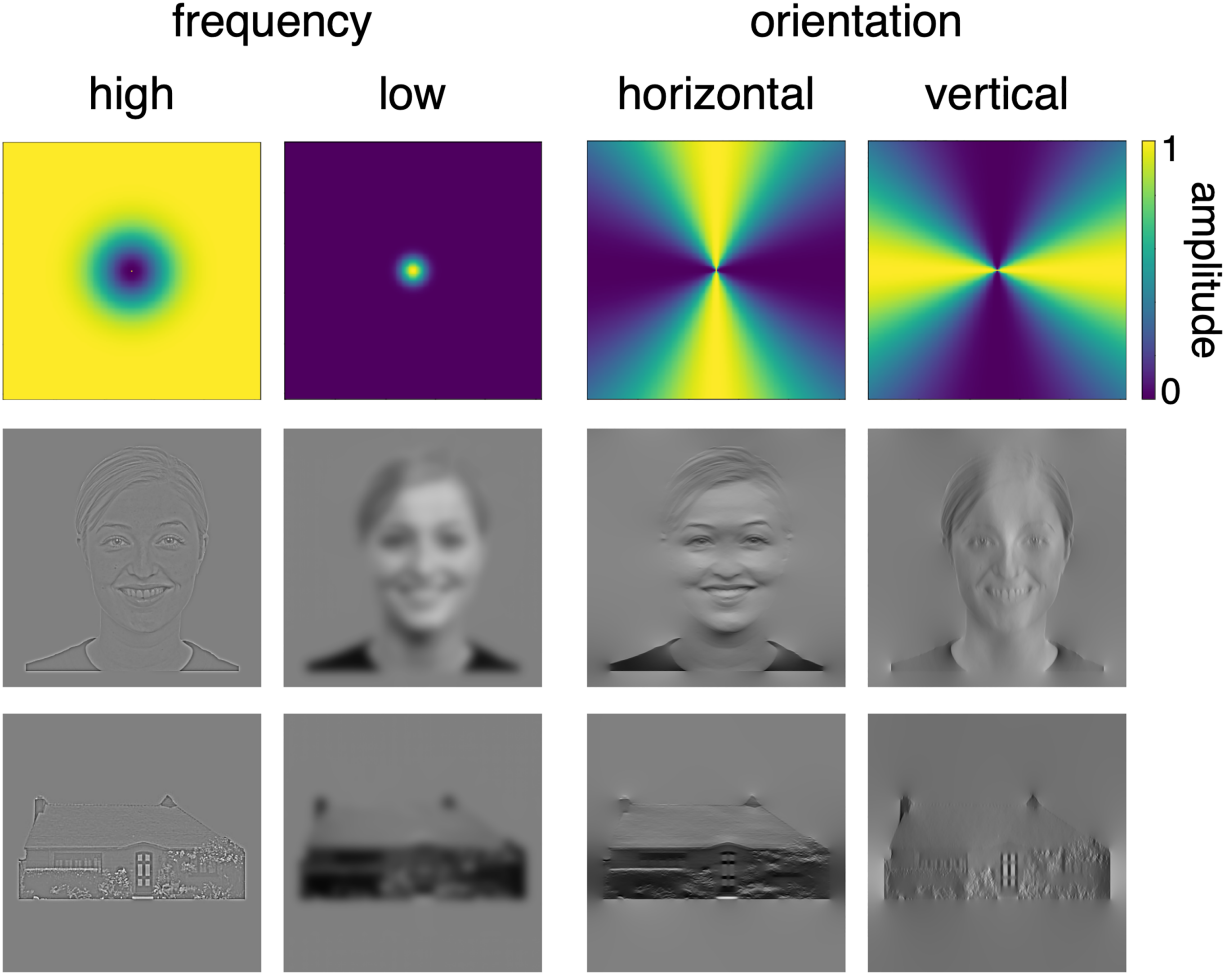
Spatial frequency and orientation filters (top) with an exemplar from the face (middle) and house (bottom) categories. The effect of high‐pass and low‐pass spatial frequency filters (left) and vertical‐pass and horizontal‐pass orientation filters (right) are shown for each exemplar

Forty‐eight face images were taken from the Radboud face database (http://www.socsci.ru.nl) and 48 house images used in previous experiments (Coggan et al., 2017; Coggan, Baker, & Andrews, 2016; Coggan, Liu, et al., 2016; Rice et al., 2014). Face images were divided across six different identities, half of which were female, and varied in viewpoint and facial expression. Filtering was performed by weighting the Fourier spectrum of each image to preserve either horizontal/vertical orientations or high/low spatial frequencies. For orientation manipulations, filters were wrapped Gaussian profiles, with a wide‐angle cut‐off (FWHM = 75°) that ensured images remained recognizable after filtering. Spatial frequency filters were Gaussian profiles with cut‐offs set at less than 2 cycles/degree and greater than 6 cycles/degree at FWHM for the low‐ and high‐pass filters respectively. Filter cut‐offs for spatial frequency were based upon those used in previous studies (Oliva & Schyns, 1997; Watson, Hymers, et al., 2016). After filtering, the global mean luminance of the images was rescaled to mid‐grey. Figure 1 shows an exemplar from each category after filters have been applied. All faces were taken from a frontal view and showed happy or neutral expressions. During the experiment, images were back‐projected onto a custom in‐bore acrylic screen and viewed via a mirror placed above the subject's head. Viewing distance was approximately 57 cm, with all images subtending approximately a 10° retinal angle. Images were presented in greyscale on a mid‐grey background. Stimulus presentation was controlled through Psychopy (Peirce et al., 2019).

**FIGURE 2:**
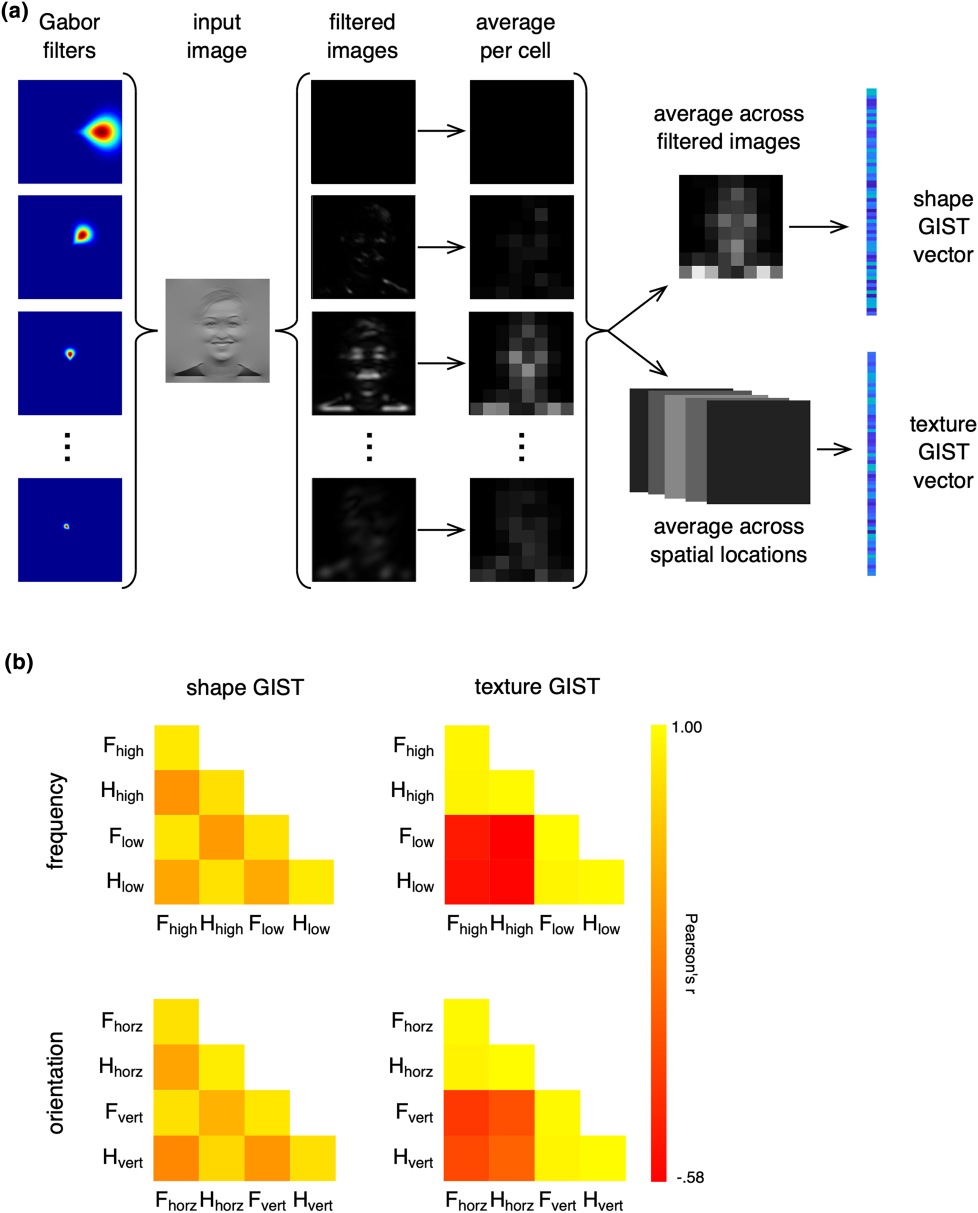
Shape and texture properties of the stimulus set. (a) Each image can be represented as a vector of values based on the low‐level properties of the image; 64 Gabor filters (shown here in Fourier space) were constructed across factorial combinations of eight spatial frequencies and eight orientations. Each filter was applied to the image in turn, resulting in 64 filtered images. Each filtered image was then windowed into an 8 × 8 grid and pixel intensities within each window were averaged. The shape GIST vector was constructed by averaging across filtered images while retaining the 8 × 8 grid, and then reshaping the resulting grid to yield a vector of 64 values. The texture GIST vector was constructed by averaging across windows for each filtered image separately, and then concatenating images to yield a vector of 64 values. (b) Similarity in shape and texture GIST vectors within and between the different conditions. Vectors were compared through correlation using a leave‐one‐image‐out paradigm. Legend for the similarity matrices: F = face, H = house, high = high spatial frequency, low = low spatial frequency, horz = horizontal, vert = vertical

To measure the ‘shape’ and ‘texture’ properties of the image, we used modified versions of the GIST descriptor (Oliva & Torralba, 2001; Watson, Hymers, et al., 2016). Each image was passed through a series of Gabor filters spanning eight orientations and eight spatial frequencies, generating 64 filtered images for each input image (Figure 2a). Next, each filtered image was divided into an 8 × 8 grid and pixel intensities were averaged within each grid cell. We then constructed two variants of the GIST descriptor that measured the shape and texture properties of each image separately.

For the shape GIST descriptor, we averaged across the filter outputs, yielding a single 8 × 8 grid that was then flattened to a vector of 64 numbers. This vector therefore represents the spatial energy across the image (higher values indicate a greater amount of energy at a specific spatial location within the image), but is insensitive to the spectral properties. For the texture GIST descriptor, each filter output was averaged over grid cells to yield a single value, and these values then concatenated into a vector of 64 numbers. This vector therefore represents the textural or nonspatial properties of the image (higher values indicate more energy at particular orientation/frequency within the image), but it is insensitive to the shape properties of the image.

The shape and texture GISTs were used to compare images across conditions with correlation (Rice et al., 2014; Watson et al., 2014; Watson, Young, & Andrews, 2016). The average correlation matrix for each GIST descriptor type (shape, texture) is shown in Figure 2b. This shows a clear difference in the shape and texture properties of the images. Images with the same filter had similar texture properties, irrespective of category. In contrast, images from the same category had similar shape properties, irrespective of filter. This manipulation allowed us to compare the relative role of shape and texture in the selectivity of responses in category‐selective regions.

## 3 EXPERIMENT 1—MVPA

### 3.1 Design and procedure

For this experiment, all 48 faces and 48 houses were passed through the four filters (two orientation, two frequency) to generate 384 images. The fMRI experiment consisted of two scans, one for each filter type (orientation, frequency). In each scan, images were presented in 9 s blocks. In each block, nine images from each category/filter combination (see Figure 1) were presented individually for 750 ms, with a 250‐ms interstimulus interval. This was followed by a fixation cross on a mid‐grey background for 9 s. Each image in the stimulus set was shown once. To maintain participants' attention, a one‐back task was used in which the participant was instructed to press a button on a response box whenever the current image was judged to be identical to the immediately previous image.

### 3.2 Regions of interest (ROIs)

**FIGURE 3:**
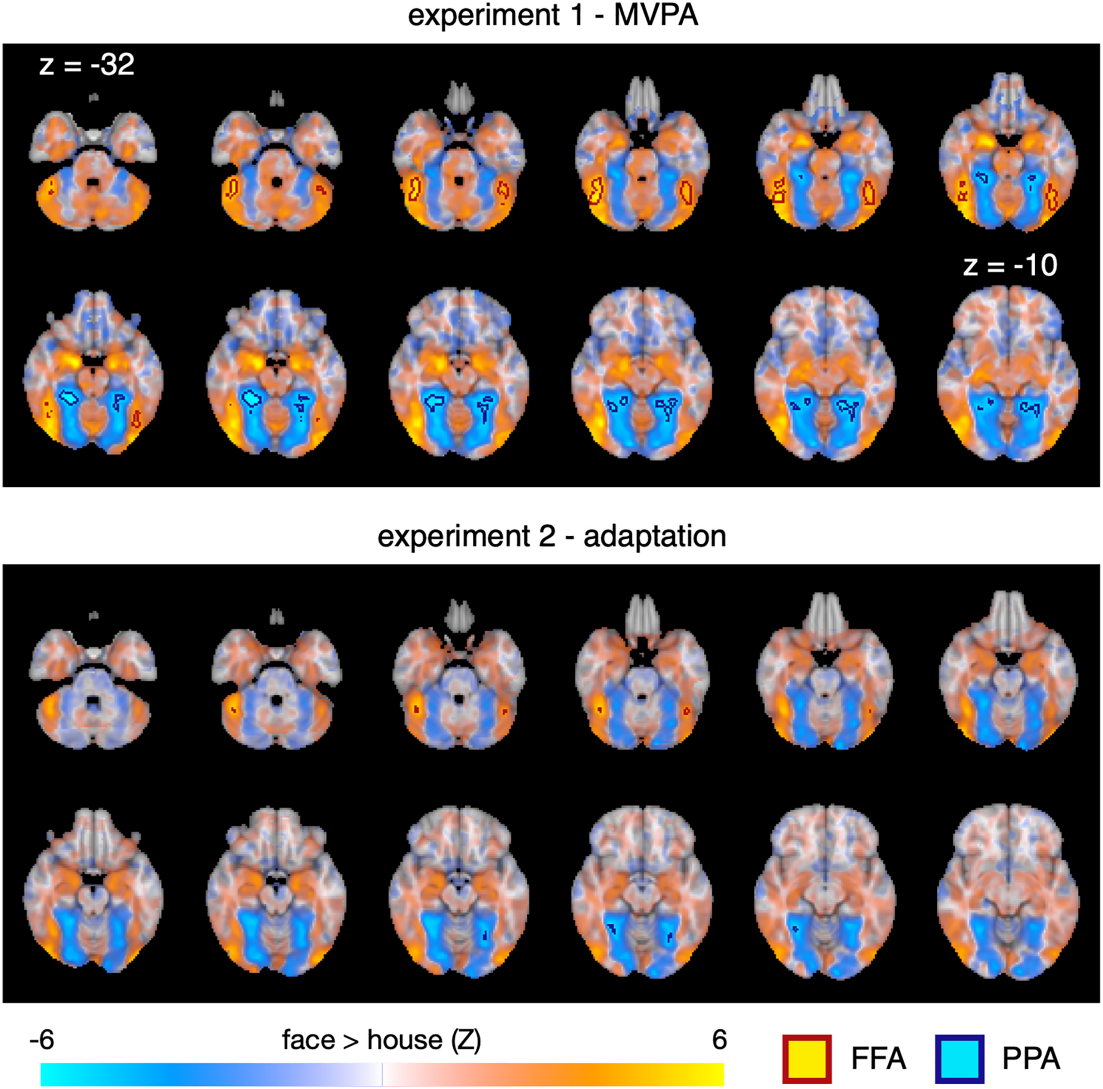
Group analysis of localizer scans in Experiment 1 and Experiment 2. The FFA and PPA were defined by the contrast between face > house and house > face, respectively.

FFA and PPA locations were identified based on an independent localizer scan performed after the experimental scans (Figure 3). Our rationale for using the FFA and PPA is that these regions are located in a similar region of ventral temporal cortex and show strong and opposing selectivity. Images were presented in a block design identical to the experimental scan. Forty‐eight face and 48 scene images were presented in 12 blocks, with each image presented once. The FFA was defined by a face > scene contrast, and the PPA was defined by a scene > face contrast. A flood‐fill algorithm was used to define group ROIs of 256 voxels per hemisphere. The algorithm works by iteratively reducing the threshold to add spatially contiguous voxels with the next highest *z* value to the cluster. This process continues until there are 256 voxels in a cluster. Due to the spatial distortion of the EPI image, the correspondence with the structural TI image is slightly misaligned. However, it is important to note the EPI data from the localizer and the experimental scans are both distorted in the same way and thus remain aligned.

### 3.3 Data analysis

The effect of shape and texture properties on patterns of neural response was tested using correlation‐based multivariate pattern analysis (Haxby et al., 2001). This was conducted independently for the spatial frequency and orientation scans. First, parameter estimates for each condition were normalized on a voxel‐wise basis for each participant. This involved subtracting the mean response across all conditions. Group analyses were then conducted with one participant being left out for each analysis. The responses from the group analyses were normalized by subtracting the mean response to all conditions. For each pairwise combination of conditions, the patterns of response in each participant were compared with a corresponding group parameter estimate from the group analysis of the remaining participants. This leave‐one‐participant‐out (LOPO) cross‐validation paradigm was repeated for each participant (Rice et al., 2014). The MVPA was implemented using the PyMVPA toolbox (Hanke et al., 2009). The correlation coefficients were then used to populate a representation similarity matrix (RSM), which shows the relative similarity of patterns of response to different conditions. A Fisher's Z‐transformation was then applied to the correlations prior to further statistical analysis. A representational similarity analysis with multiple regression was used to determine the role of shape and texture (Watson, Hymers, et al., 2016; Watson, Young, & Andrews, 2016). This involved using the shape and texture GIST correlation matrices from Figure 2b as regressors, while the fMRI MVPA correlation matrices for each participant were entered as the outcome variable.

## 4 EXPERIMENT 2—FMR‐ADAPTATION

### 4.1 Design and procedure

Six faces and six houses from each category were used in this experiment. The faces included three male and three female identities that were front facing and were smiling. These images were passed through four different filters (two orientation, two frequency) to give a total of 48 different images. There were two fMRI scans with images filtered by orientation and frequency presented in different scans. The order of scans was counterbalanced across subjects. In each scan, images were presented in 6‐s blocks. In each block, six images were presented individually for 800 ms, with a 200‐ms interstimulus interval. This was followed by a fixation cross lasting 9 s.

The fMR‐adaptation had three different image sequences: no change; shape change; texture change (see Figure 5a). These image sequences were shown for each object category. The advantage of using a limited number of exemplars is that the same images are used in different conditions and it is only the sequence of images that is different. Each combination of filter, category and sequence was shown in six blocks. The order of the blocks was randomized for each subject and scan. Participants performed a task that consisted of pressing a button on a response box whenever the fixation cross during the inter‐stimulus interval was shown in green while viewing images. The task was designed to maintain attention for the duration of the scan. Green fixation crosses were randomly placed after 36 of the 216 images presented throughout each scan. Analysis of variance (ANOVA) and post‐hoc t‐tests were used to determine the effect of shape and texture properties on the response to faces and houses. Benjamini–Hochberg correction for multiple comparisons was applied across comparisons for each ROI (Benjamini & Hochberg, 1995).

To determine if participants were able to discriminate a change in filter from a change in exemplar, we ran a behavioural experiment outside the scanner using the same stimulus conditions. Participants (*n* = 10, 5 male, median/min/max age = 23/19/28, SD = 3.0 years) were asked to indicate if two images were from the same identity (change in filter) or from a different identity (change in exemplar). For spatial frequency trials, participants were accurate on 87.0 ± 0.03% (mean ± sem) with a *d*′ = 3.04. For orientation trials, participants were accurate on 85.0 ± 0.04% (mean ± sem) of Orientation filtered trials with a *d*′ = 3.04.

### 4.2 ROIs

FFA and PPA locations (Figure 3) were identified based on a group analysis of the experimental fMRI data. First, two parameter estimates were generated, combining all face or all house conditions. The peak face‐ and house‐selective voxels (i.e., those with the highest z value) were identified using face > house and house > face contrasts, respectively. For the peak FFA and PPA voxels in left and right hemispheres, a flood fill algorithm was used to identify a cluster of 16 spatially contiguous voxels for each ROI.

### 4.3 Data analysis

Functional time‐series of responses were collapsed across voxels in each ROI and converted to percent signal change for each participant. The time‐series from each ROI was then divided into six TR (18 s) stimulus blocks. The data were normalized by subtracting a baseline (calculated as the mean of the six TR values from each block) from each value within a block. Blocks from the same stimulus condition were averaged to produce a mean time‐series. The peak neural responses at TR 3 (9 s, post‐stimulus onset) were entered into a four‐way ANOVA with ROI (FFA, PPA), Filter (frequency, orientation), Category (face, house) and Sequence (no change; shape change; texture change) as repeated measures.

### 4.4 Data acquisition

All fMRI data were acquired with a General Electric 3T HD Excite MRI scanner in YNiC at the University of York, fitted with an eight‐channel, phased‐array, head‐dedicated gradient insert coil tuned to 127.4 MHz. A gradient‐echo echo‐planar imaging (EPI) sequence was used to collect data from 38 contiguous axial slices (TR = 3000 ms, TE = 32.7 ms, FOV = 288 × 288 mm, matrix size = 128 × 128, voxel dimensions = 2.25 × 2.25 × 3 mm, flip angle = 90°). The fMRI data were analysed with FEAT v5.98 (http://www.fmrib.ox.ac.uk/fsl). In all scans, the initial 9 s of data were removed to reduce the effects of magnetic saturation. Motion correction (MCFLIRT, FSL) and slice‐timing correction were applied, followed by temporal high‐pass filtering (Gaussian‐weighted least‐squares straight line fitting, sigma = 50 s). Gaussian spatial smoothing was applied at 6‐mm FWHM. Parameter estimates were generated for each cluster by regressing the hemodynamic response of each voxel against a box‐car function convolved with a single‐gamma HRF. Functional data were first registered to a low‐resolution T1‐anatomical image oriented in the same plane as the EPI (TR = 2.5 s, TE = 9.98 ms, FOV = 288 × 288 mm, matrix size = 512 × 512, voxel dimensions = 0.56 × 0.56 × 3 mm, flip angle = 90°), then to a high‐resolution T1‐anatomical image (TR = 7.96 ms, TE = 3.05 ms, FOV = 290 × 290 mm, matrix size = 256 × 256, voxel dimensions = 1.13 × 1.13 × 1 mm, flip angle = 20°) and finally onto the standard MNI brain (ICBM152).

An arbitrary alpha value of 0.05 was used to indicate significant effects.

## 5 RESULTS

### 5.1 Experiment 1—MVPA

**FIGURE 4:**
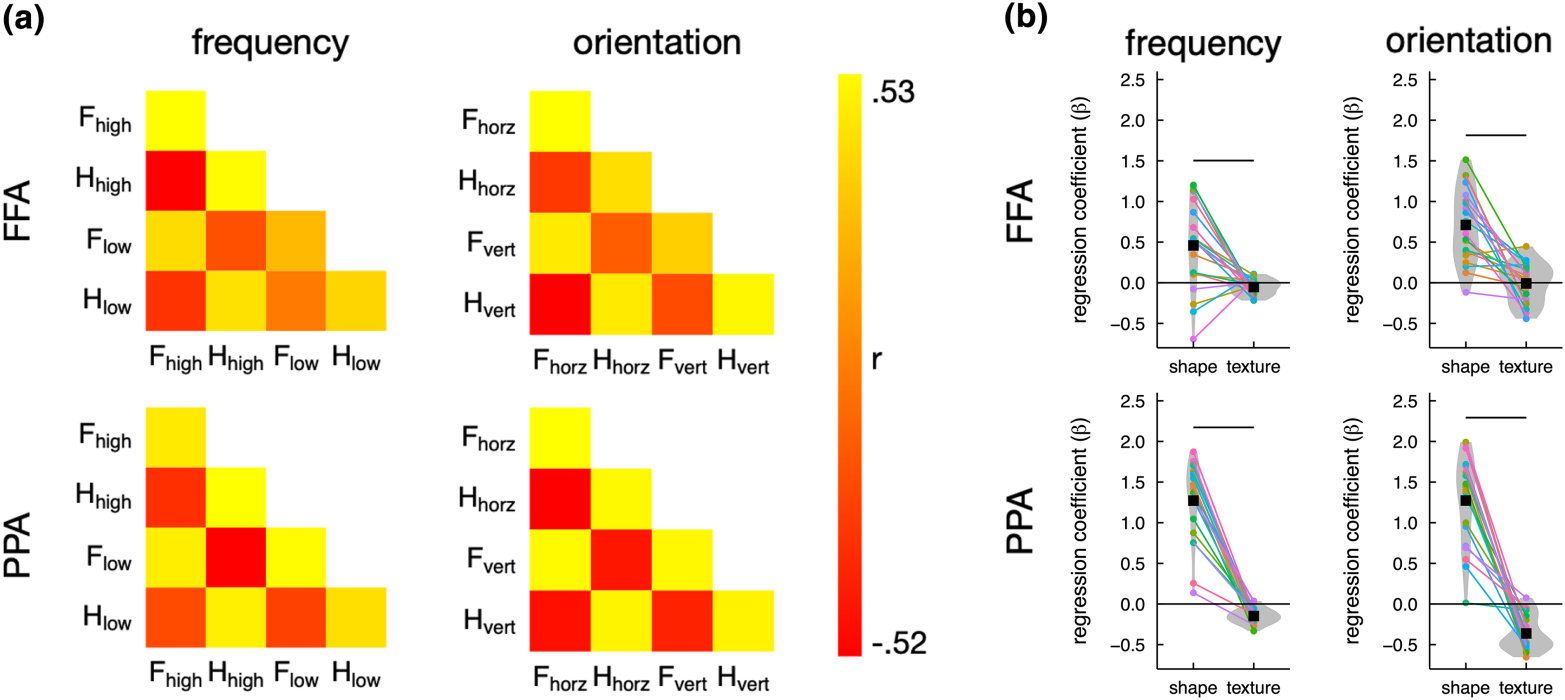
Experiment 1—MVPA. (a) Similarity matrices in the FFA and PPA (F = face, H = house, high = high spatial frequency, low = low spatial frequency, horz = horizontal, vert = vertical). (b) Coefficients for the shape and texture GIST models (see Figure 2b) in a multiple regression of the fMRI similarity matrices. All regressors and outcomes were Z‐scored such that coefficients are given in standardised units. Error bars represent standard error of the mean. The pattern of response was predicted by the shape rather than the texture GIST model. Horizontal line indicates *p* < 0.05

The similarity in the patterns of response across conditions in the FFA and PPA is shown in Figure 4a. To determine the role of shape and texture properties on the patterns of neural response, we used a representational similarity analysis with multiple regression (Watson, Hymers, et al., 2016; Watson, Young, & Andrews, 2016). The shape and texture GIST correlation matrices from Figure 2b were entered as regressors, while the fMRI MVPA correlation matrices for each participant, were entered as the outcome variable. The coefficient for each regressor is shown in Figure 4b.

In the FFA, the shape GIST significantly predicted responses in both the frequency scan (*β* = 0.46, *t*(19) = 3.74, Cohen's *d*_*z*_ = 0.84, *p* = 0.002) and the orientation scan (*β* = 0.71, *t*(19) = 6.98, Cohen's *d*_*z*_ = 1.56, *p* < 0.001). In contrast, the texture GIST coefficient was significantly below zero for the frequency scan (*β* = −0.05, *t*(19) = 2.44, Cohen's *d*_*z*_ = 0.54, *p* = 0.027) and not significantly different from zero in the orientation scan (*β* − 0.01, *t*(19) = 0.15, Cohen's *d*_*z*_ = 0.03, *p* = 0.881). The shape GIST coefficient was significantly greater than the texture GIST coefficient in both scans (frequency: *t*(19) = 3.72, Cohen's *d*_*z*_ = 1.30, *p* = 0.002; orientation: *t*(19) = 5.69, Cohen's *d*_*z*_ = 1.97, *p* < 0.001).

In the PPA, the shape GIST significantly predicted responses in the frequency scan (*β* = 1.27, *t*(19) = 11.66, Cohen's *d*_*z*_ = 2.61, *p* < 0.001) and the orientation scan (*β* = 1.27, *t*(19) = 10.29, Cohen's *d*_*z*_ = 2.30, *p* < 0.001). Conversely, the texture GIST coefficient was significantly below zero for both frequency (*β* = −0.15, *t*(19) = −6.88, Cohen's *d*_*z*_ = 1.54, *p* < 0.001) and orientation (*β* = −0.36, *t*(19) = 7.61, Cohen's *d*_*z*_ = 1.70, *p* = 0.129). The shape GIST coefficient was significantly greater than the texture GIST coefficient in both scans (frequency: *t*(19) = 12.62, Cohen's *d*_*z*_ = 4.04, *p* < 0.001; orientation: *t*(19) = 10.51, Cohen's *d*_*z*_ = 3.90, *p* < 0.001).

We also analysed responses in other known face‐ and scene‐selective regions in visual cortex. This included the face‐selective occipital face area (OFA) and posterior superior temporal sulcus (pSTS) and the scene‐selective occipital place area (OPA) and retrosplenial complex (RSC). Table S1 shows that the patterns of response in these category‐selective regions were more strongly predicted by the shape model than the texture model in both the orientation and spatial frequency scans.

To understand how the representation in category‐selective regions emerges, we also analysed regions in the occipital and temporal cortices using probabilistic maps of visual topography (Wang et al., 2015). The performance of the shape and texture models in these regions is shown in Table S2. Although there was a general bias towards the shape model compared with the texture model, the pattern of neural response was predicted by the texture model in a number of these regions. However, this was only for the frequency scans, with some of the ventral regions (V1v, V2d and hV4) showing no difference between the shape and texture models.

### 5.2 Experiment 2

**FIGURE 5:**
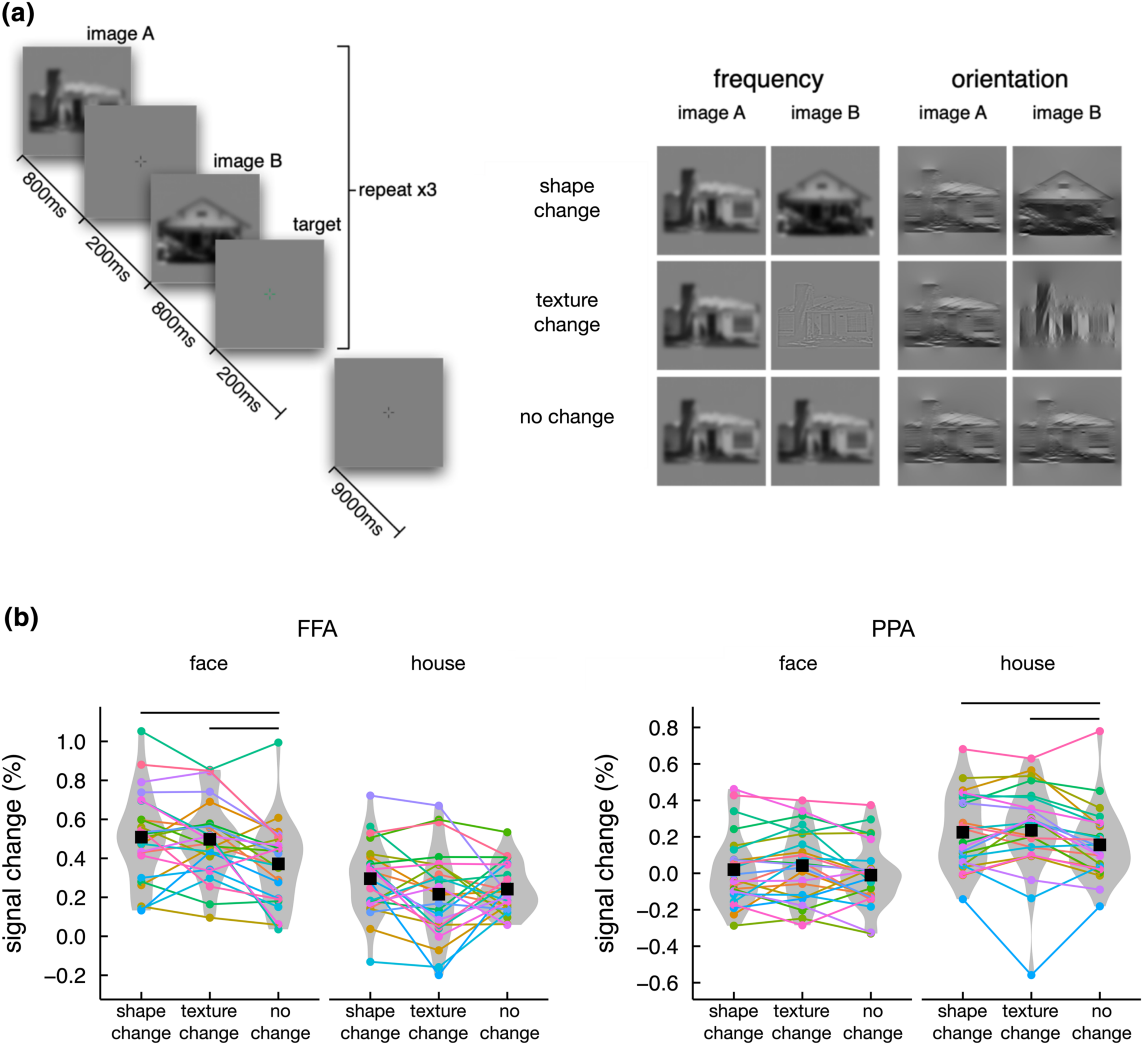
Experiment 2. (a) Stimulus presentation sequences for house stimuli. There were three different stimulus conditions: No change; shape change; texture change. These conditions were presented using images manipulated by spatial frequency or orientation. Pairs of images in each condition were presented sequentially and repeated three times to give a total of six images in each block. (b) Response in FFA and PPA to different conditions. Data were collapsed across frequency and orientation filters. Error bars represent standard error of the mean. Higher responses were evident to the texture change and shape change conditions compared with the same exemplar, same filter condition for the preferred, but not the nonpreferred stimulus in each region. Horizontal line indicates *p* < 0.05

fMR‐adaptation was used to determine the sensitivity in the FFA and PPA to sequences of faces or houses in which there was either a change in exemplar or a change in filter (Figure 5a). First, we asked whether there was any difference in the choice of filter (orientation or spatial frequency). A four‐way, repeated‐measures ANOVA (Filter, ROI, Category, Sequence) revealed no main effect or interactions involving Filter (Filter * ROI: *F*(1,23) = 3.75, *p* = 0.065; Filter * Category: *F*(1,23) = 2.22, *p* = 0.150; Filter * Sequence: *F*(2,46) = 0.034, *p* = 0.966; Filter * ROI * Category: *F*(1,23) = 1.48, *p* = 0.237; Filter * ROI * Sequence: *F*(1,23) = 0.03, *p* = 0.969; Filter * Category * Sequence: *F*(2,46) = 0.52, *p* = 0.599; Filter * ROI * Category * Sequence: *F*(2,46) = 0.25, *p* = 0.780), so all subsequent data were collapsed across Filter.

Figure 5b (Figure S1) shows the response in the FFA and PPA to different conditions (no change, shape change, texture change) with faces and houses. There was a significant three‐way interaction between ROI, category and sequence (*F*(2,46) = 13.93, *η*_G_^2^ = 0.005, *p* < 0.001). This suggests that different sequences had varying effects on the neural responses across the FFA and PPA, depending on which category was presented. To explore this, we conducted 12 planned pairwise comparisons: For each combination of ROI and category, all three sequences were contrasted against one another.

In the FFA, the response to faces was significantly lower (indicating adaptation) for no change condition compared with both shape change (*t*(23) = 3.02, Cohen's *d*_*z*_ = 0.62, *p* = 0.018) and texture change (*t*(23) = 3.65, Cohen's *d*_*z*_ = 0.75, *p* = 0.008) conditions. However, there was no significant difference (indicating a similar release from adaptation) in the response to faces between shape change and texture change conditions (*t*(23) = 0.37, Cohen's *d*_*z*_ = 0.08, *p* = 0.713). Finally, there was no significant adaptation across the different house conditions (for all comparisons, *t*(23) < 2.22, Cohen's *d*_*z*_ < 0.46, *p* > 0.07). This shows a comparable face‐selective release from adaptation in the FFA when either the shape or texture properties are changed.

In the PPA, the neural response to houses was significantly lower (indicating adaptation) for no change condition compared with the shape change condition (*t*(23) = 3.14, Cohen's *d*_*z*_ = 0.64, *p* = 0.028). The response to texture change condition was significantly higher than the no change condition for houses (*t*(23) = 2.61, Cohen's *d*_*z*_ = 0.53, *p* = 0.046). There was also no difference (indicating a similar release from adaptation) between shape change and texture change (*t*(23) = 0.39, Cohen's *d*_*z*_ = 0.08, *p* = 0.701) house conditions. Finally, there were no significant differences in the neural response across the sequences of faces (for all comparisons, *t*(23) < 1.90, Cohen's *d*_*z*_ < 0.39, *p* > 0.07). This shows a comparable house‐selective release from adaptation in the PPA when either the shape or texture properties are changed.

Again, we tested responses in other face‐selective and scene‐selective regions (OFA, STS, OPA, RSC). There was no effect of Sequence or any interaction between Sequence and Category in any of the regions (Table S3). In the visual field regions (Table S4), an effect of Sequence was found in V4 (*F*(2,46) = 10.88, *p* = 0.002), V3v (*F*(2,46) = 4.17, *p* = 0.022) and V01 (*F*(2,46) = 7.39, *p* = 0.002). However, there was no interaction between Sequence and Category. This suggests that the category‐specific fMR‐adaptation shown by the interaction between Sequence and Category emerges in the FFA and PPA.

## 6 DISCUSSION

Understanding the image properties that are important for the perception and recognition of objects is a central goal in vision science. To this end, an important distinction has been made between the role of shape and texture properties. As objects vary in both shape and texture, both sources of information could contribute to perception. Some studies have suggested the primacy of shape properties or the spatial envelope in object recognition (Biederman, 1987; Grill‐Spector et al., 1998; Malach et al., 1995; Op de Beeck et al., 2008). However, other studies have shown that texture properties can also provide important information for the perception and recognition of objects (Andrews et al., 2016; Cant et al., 2009; Cant & Goodale, 2007, 2011; Cavina‐Pratesi et al., 2010). The aim of this study was to investigate how shape and texture properties are represented in category‐selective regions of ventral temporal cortex. Using MVPA and fMR‐adaptation, we asked whether the selectivity of response in the FFA and PPA to faces and houses, respectively, is due to differences in the shape or texture properties of the stimulus.

Shape and texture properties were manipulated by applying orientation or frequency filters to the images. To measure the effect of filter, we generated modified versions of the GIST descriptor (Oliva & Torralba, 2001): One version of the GIST descriptor was sensitive to shape properties of the stimulus (shape GIST), while the other version was sensitive to nonshape properties, such as orientation and spatial frequency content (texture GIST). Filtering had a marked effect on the texture properties, but it had little effect on the shape properties of the images (see Figure 2).

We then measured the patterns of response to filtered images of faces and houses using MVPA. We found that the pattern of response to faces and houses in the FFA and PPA was predicted by the shape properties, but not by the texture properties, of the images. The importance of the shape properties of the image in predicting patterns of response in high‐level visual cortex fits with other studies using MVPA (Bracci & Op de Beeck, 2016; Op de Beeck et al., 2008; Vernon et al., 2016; Watson, Young, & Andrews, 2016). For example, we showed that patterns of response to different objects in ventral temporal cortex was predicted more by shape properties than by texture properties (Watson, Young, & Andrews, 2016). However, our current findings show some differences to other studies, which have shown an effect of texture properties on patterns of response in the PPA (Berman et al., 2017; Lowe et al., 2016, 2017; Watson, Hymers, et al., 2016). These studies show that the frequency content of the image does influence the pattern of response. The differences between the current study and these previous studies are likely to reflect the images we have used. Here, we showed isolated images of faces and houses superimposed on a uniform background, while these other studies showed images of scenes that encompassed the full extent of the display. Therefore, in our study, significant differences in the spatial extent of the images remain after they have been passed through a filter (see Figure 1). It seems that these spatial differences play a dominant role in differentiating patterns of response to isolated faces and houses.

Next, we used fMR‐adaptation to measure the response to faces and houses. We found a release from adaptation to images that differed in texture properties. This is consistent with previous studies that have shown sensitivity to texture properties in high‐level visual cortex (Andrews et al., 2016; Cant et al., 2009; Cant & Goodale, 2007, 2011). However, the release from adaptation caused by changing the texture properties was only evident for the preferred category (FFA: faces, PPA: houses). Although this implies a greater sensitivity to the properties of the preferred stimulus (Andrews et al., 2010), it is important to note that this difference could be explained by the lower response to the nonpreferred stimulus. Our findings are consistent with other studies using fMR‐adaptation that have shown a release from adaptation to changes in the image of the same exemplar (Andrews & Ewbank, 2004; Byrne et al., 2016; Davies‐Thompson et al., 2009, 2013; Eger et al., 2004; Ewbank et al., 2005; Grill‐Spector et al., 1999; Lowe et al., 2017; O'Connell & Chun, 2018; Pourtois et al., 2005a, 2005b). For example, O'Connell et al. (2018) showed in the PPA that successive presentations of two line drawings or two photographs of the same scene resulted in a reduced response (adaptation) compared with the presentation of two different scenes. However, when a line drawing and then a photograph of the same scene were presented in sequence, thus changing the texture properties, there was a release from adaptation (higher response).

The difference in the sensitivity to filtering between the fMR‐adaptation and MVPA analyses provides information about the scale at which shape and texture information is represented in the FFA and PPA. Because MVPA reveals patterns of response across voxels it provides coarser‐scale information about neural representation than is evident at the single voxel level (Haxby et al., 2014; Tong & Pratte, 2012). There has been some debate about the spatial scale of the pattern of response in MVPA (cf Freeman et al., 2011; Kamitani & Tong, 2005). Our MVPA method compared the pattern of response in each participant with the pattern from the group (with that participant left out). This leave one participant out (LOPO) approach has been used in previous studies shows that patterns of response are consistent across participants (Coggan, Liu, et al., 2016; Flack et al., 2015; Rice et al., 2014; Watson et al., 2014; Weibert et al., 2018). However, because of the need for registering patterns of response across different brains, this will only reveal a coarse scale representation. Because the MVPA experiment showed that the pattern of neural response was predicted by the shape properties of the images, but not by their texture properties, this implies that shape information is represented is represented at a coarser level. In contrast, fMR‐adaptation can detect finer‐scale neural responses within voxels (Andrews, 2005; Avidan et al., 2002; Epstein & Morgan, 2012). Therefore, the sensitivity to the texture properties of the images in the fMR‐adaptation experiment suggests that it is dependent on a finer scale of representation.

A number of studies have shown that differences between textures can also be perceived from the second order differences and that selectivity to these differences emerges at later stages of processing (Coggan et al., 2017; Freeman et al., 2013; Freeman & Simoncelli, 2011). More recent reports have begun to directly uncover the relationship between textural properties of the image and physical surfaces in the real world (Fleming & Storrs, 2019; Schmid et al., 2021). Our findings suggest that future studies that attempt to uncover the neural correlates of surface properties will need to investigate neural responses at this finer scale of representation.

In conclusion, these findings reveal novel insights into the way that shape and texture properties of objects are represented in category‐selective regions and demonstrate the importance of combining MVPA and fMR‐adaptation paradigms to understand how information is represented in ventral temporal cortex across different neuronal scales (Drucker & Aguirre, 2009; Hatfield et al., 2016; O'Connell & Chun, 2018).

## CONFLICT OF INTEREST

The authors declare no competing financial interests.

## AUTHOR CONTRIBUTIONS

DDC, DMW and TJA conceived and designed the experiments. DDC, DMW, AW, RB, CE, KJ and CK collected and analysed the data. DDC and TJA wrote the initial draft of the manuscript. All authors contributed to the submitted manuscript.

### PEER REVIEW

The peer review history for this article is available at https://publons.com/publon/10.1111/ejn.15737.

## Supporting information


**Table S1.** Results for additional face‐ and scene‐selective regions in experiment 1. p values were corrected for multiple comparisons using a Benjamini‐Hochberg correction across 36 comparisons (6 ROIs including FFA and PPA, 2 filter types, 3 model contrasts). * p < 0.05
**Table S2.** Results for retinotopic regions in experiment 1. p values were corrected for multiple comparisons using a Benjamini‐Hochberg correction across 150 comparisons (25 ROIs, 2 filter types, 3 model contrasts). * p < 0.05
**Table S3.** Results for additional face‐ and scene‐selective regions in experiment 2. Shape adaptation is defined as shape change – no change; texture adaptation is defined as texture change – no change. p values were corrected for multiple comparisons using a Benjamini‐Hochberg correction across 6 comparisons for each ROI/filter combination. * p < 0.05
**Table S4.** Results for retinotopic regions in experiment 2. p values were corrected for multiple comparisons using a Benjamini‐Hochberg correction across 6 comparisons in each ROI/filter combination. * p < 0.05
**Figure S1.** Response in FFA and PPA to different conditions from experiment 2. Data were separated across frequency and orientation filters

## Data Availability

Data and code are available at https://github.com/ddcoggan/p008.
