# Supplementary material for: The representation of shape and texture in category‐selective regions of ventral‐temporal cortex
Source: Eur J Neurosci. 2022 Jun 21;56(3):4107–20. doi: 10.1111/ejn.15737 (PMC9545892; doi:10.1111/ejn.15737)
Supplement: Supplementary file 1 — Table S1. Results for additional face‐ and scene‐selective regions in experiment 1. p values were corrected for multiple comparisons using a Benjamini‐Hochberg correction across 36 comparisons (6 ROIs including FFA and PPA, 2 filter types, 3 model contrasts). * p < 0.05 Table S2. Results for retinotopic regions in experiment 1. p values were corrected for multiple comparisons using a Benjamini‐Hochberg correction across 150 comparisons (25 ROIs, 2 filter types, 3 model contrasts). * p < 0.05 Table S3. Results for additional face‐ and scene‐selective regions in experiment 2. Shape adaptation is defined as shape change – no change; texture adaptation is defined as texture change – no change. p values were corrected for multiple comparisons using a Benjamini‐Hochberg correction across 6 comparisons for each ROI/filter combination. * p < 0.05 Table S4. Results for retinotopic regions in experiment 2. p values were corrected for multiple comparisons using a Benjamini‐Hochberg correction across 6 comparisons in each ROI/filter combination. * p < 0.05 Figure S1. Response in FFA and PPA to different conditions from experiment 2. Data were separated across frequency and orientation filters [file EJN-56-4107-s001.docx]

| Region | Filter | Model | Mean β | SEM β | t | d_z_ | p |
| --- | --- | --- | --- | --- | --- | --- | --- |
| OFA | frequency | shape | 0.73 | 0.11 | 6.8 | 1.52 | <.0001* |
|  |  | texture | -0.08 | 0.02 | -3.93 | 0.88 | .0013* |
|  |  | shape - texture | -0.81 | 0.11 | -7.48 | 2.35 | <.0001* |
|  | orientation | shape | 0.79 | 0.1 | 7.59 | 1.7 | <.0001* |
|  |  | texture | -0.17 | 0.05 | -3.57 | 0.8 | .0026* |
|  |  | shape - texture | -0.96 | 0.12 | -8.24 | 2.65 | <.0001* |
| pSTS | frequency | shape | 0.69 | 0.1 | 7.2 | 1.61 | <.0001* |
|  |  | texture | -0.16 | 0.04 | -4.25 | 0.95 | .0007* |
|  |  | shape - texture | -0.85 | 0.1 | -8.72 | 2.61 | <.0001* |
|  | orientation | shape | 0.84 | 0.09 | 9 | 2.01 | <.0001* |
|  |  | texture | -0.21 | 0.05 | -4.17 | 0.93 | .0008* |
|  |  | shape - texture | -1.05 | 0.11 | -9.55 | 3.13 | <.0001* |
| OPA | frequency | shape | 0.68 | 0.12 | 5.74 | 1.28 | <.0001* |
|  |  | texture | -0.03 | 0.02 | -1.46 | 0.33 | .1701 |
|  |  | shape - texture | -0.71 | 0.12 | -5.77 | 1.87 | <.0001* |
|  | orientation | shape | 0.74 | 0.12 | 6.1 | 1.36 | <.0001* |
|  |  | texture | -0.17 | 0.06 | -3.14 | 0.7 | .0065* |
|  |  | shape - texture | -0.91 | 0.14 | -6.51 | 2.17 | <.0001* |
| RSC | frequency | shape | 0.11 | 0.11 | 0.97 | 0.22 | .3518 |
|  |  | texture | -0.22 | 0.07 | -3.19 | 0.71 | .0059* |
|  |  | shape - texture | -0.33 | 0.11 | -3.07 | 0.78 | .0073* |
|  | orientation | shape | 0.45 | 0.11 | 4.16 | 0.93 | .0008* |
|  |  | texture | -0.22 | 0.09 | -2.44 | 0.55 | .0271* |
|  |  | shape - texture | -0.67 | 0.1 | -6.56 | 1.5 | <.0001* |

**Supplementary Table 1.** Results for additional face- and scene-selective regions in experiment 1. p values were corrected for multiple comparisons using a Benjamini-Hochberg correction across 36 comparisons (6 ROIs including FFA and PPA, 2 filter types, 3 model contrasts). * p < .05

| Region | Filter | Model | Mean β | SEM β | t | d_z_ | p |
| --- | --- | --- | --- | --- | --- | --- | --- |
| V1d | frequency | shape | 0.42 | 0.10 | 4.00 | 0.89 | .0013* |
|  |  | texture | -0.01 | 0.04 | -0.32 | 0.07 | .7495 |
|  |  | shape - texture | -0.43 | 0.11 | -3.90 | 1.23 | .0016* |
|  | orientation | shape | 0.60 | 0.13 | 4.50 | 1.01 | .0001* |
|  |  | texture | -0.17 | 0.05 | -3.18 | 0.71 | .0068* |
|  |  | shape - texture | -0.77 | 0.14 | -5.38 | 1.70 | .0001* |
| V2d | frequency | shape | 0.30 | 0.10 | 3.02 | 0.68 | .0089* |
|  |  | texture | 0.08 | 0.03 | 3.10 | 0.69 | .0080* |
|  |  | shape - texture | -0.21 | 0.09 | -2.31 | 0.66 | .0358* |
|  | orientation | shape | 0.13 | 0.12 | 1.05 | 0.24 | .3124 |
|  |  | texture | -0.20 | 0.08 | -2.58 | 0.58 | .0215* |
|  |  | shape - texture | -0.33 | 0.09 | -3.64 | 0.72 | .0026* |
| V3d | frequency | shape | 0.55 | 0.13 | 4.14 | 0.93 | .0010* |
|  |  | texture | -0.11 | 0.03 | -3.75 | 0.84 | .0022* |
|  |  | shape - texture | -0.65 | 0.13 | -5.17 | 1.53 | .0001* |
|  | orientation | shape | 0.75 | 0.09 | 8.30 | 1.86 | <.0001* |
|  |  | texture | -0.19 | 0.07 | -2.78 | 0.62 | .0143* |
|  |  | shape - texture | -0.94 | 0.15 | -6.43 | 2.61 | <.0001* |
| V3a | frequency | shape | 0.64 | 0.11 | 5.54 | 1.24 | .0001* |
|  |  | texture | -0.03 | 0.03 | -0.94 | 0.21 | .3612 |
|  |  | shape - texture | -0.66 | 0.12 | -5.31 | 1.77 | .0001* |
|  | orientation | shape | 0.29 | 0.14 | 1.98 | 0.44 | .0669 |
|  |  | texture | -0.18 | 0.05 | -3.75 | 0.84 | .0022* |
|  |  | shape - texture | -0.46 | 0.15 | -3.18 | 0.96 | .0068* |
| V3b | frequency | shape | 0.48 | 0.13 | 3.61 | 0.81 | .0027* |
|  |  | texture | -0.06 | 0.04 | -1.83 | 0.41 | .0892 |
|  |  | shape - texture | -0.55 | 0.15 | -3.66 | 1.25 | .0026* |
|  | orientation | shape | 0.36 | 0.14 | 2.61 | 0.58 | .0205* |
|  |  | texture | -0.15 | 0.06 | -2.45 | 0.55 | .0275* |
|  |  | shape - texture | -0.51 | 0.18 | -2.82 | 1.06 | .0132* |
| LO1 | frequency | shape | 0.44 | 0.10 | 4.53 | 1.01 | .0005* |
|  |  | texture | -0.06 | 0.03 | -2.39 | 0.54 | .0303* |
|  |  | shape - texture | -0.50 | 0.10 | -4.91 | 1.58 | .0002* |
|  | orientation | shape | 0.56 | 0.10 | 5.81 | 1.30 | <.0001* |
|  |  | texture | -0.14 | 0.05 | -2.95 | 0.66 | .0103* |
|  |  | shape - texture | -0.70 | 0.13 | -5.28 | 2.06 | .0001* |
| LO2 | frequency | shape | 0.58 | 0.16 | 3.66 | 0.82 | .0026* |
|  |  | texture | -0.08 | 0.03 | -2.98 | 0.67 | .0096* |
|  |  | shape - texture | -0.66 | 0.17 | -3.97 | 1.30 | .0014* |
|  | orientation | shape | 0.67 | 0.12 | 5.66 | 1.27 | .0001* |
|  |  | texture | -0.27 | 0.06 | -4.37 | 0.98 | .0007* |
|  |  | shape - texture | -0.94 | 0.16 | -5.92 | 2.22 | <.0001* |
| hMT | frequency | shape | 0.91 | 0.18 | 4.95 | 1.11 | .0002* |
|  |  | texture | -0.11 | 0.04 | -2.55 | 0.57 | .0222* |
|  |  | shape - texture | -1.03 | 0.19 | -5.46 | 1.71 | .0001* |
|  | orientation | shape | 0.87 | 0.13 | 6.75 | 1.51 | <.0001* |
|  |  | texture | -0.22 | 0.08 | -2.86 | 0.64 | .0124* |
|  |  | shape - texture | -1.10 | 0.17 | -6.28 | 2.29 | <.0001* |
| V1v | frequency | shape | 0.26 | 0.10 | 2.56 | 0.57 | .0221* |
|  |  | texture | -0.14 | 0.05 | -3.09 | 0.69 | .0080* |
|  |  | shape - texture | -0.40 | 0.12 | -3.41 | 1.14 | .0042* |
|  | orientation | shape | 0.74 | 0.17 | 4.25 | 0.95 | .0008* |
|  |  | texture | -0.22 | 0.07 | -3.14 | 0.70 | .0073* |
|  |  | shape - texture | -0.95 | 0.21 | -4.50 | 1.61 | .0005* |
| V2v | frequency | shape | 0.47 | 0.13 | 3.51 | 0.78 | .0034* |
|  |  | texture | -0.17 | 0.06 | -3.07 | 0.69 | .0082* |
|  |  | shape - texture | -0.64 | 0.14 | -4.62 | 1.39 | .0004* |
|  | orientation | shape | 1.14 | 0.15 | 7.41 | 1.66 | <.0001* |
|  |  | texture | -0.32 | 0.07 | -4.36 | 0.98 | .0007* |
|  |  | shape - texture | -1.46 | 0.20 | -7.43 | 2.71 | <.0001* |
| V3v | frequency | shape | 0.60 | 0.14 | 4.35 | 0.97 | .0007* |
|  |  | texture | -0.17 | 0.04 | -3.88 | 0.87 | .0017* |
|  |  | shape - texture | -0.76 | 0.12 | -6.42 | 1.68 | <.0001* |
|  | orientation | shape | 1.05 | 0.14 | 7.45 | 1.67 | <.0001* |
|  |  | texture | -0.34 | 0.08 | -4.34 | 0.97 | .0007* |
|  |  | shape - texture | -1.38 | 0.14 | -9.78 | 2.73 | <.0001* |
| hV4 | frequency | shape | 0.28 | 0.09 | 3.09 | 0.69 | .0080* |
|  |  | texture | 0.07 | 0.05 | 1.44 | 0.32 | .1721 |
|  |  | shape - texture | -0.21 | 0.10 | -2.06 | 0.62 | .0585 |
|  | orientation | shape | 0.56 | 0.15 | 3.71 | 0.83 | .0024* |
|  |  | texture | -0.12 | 0.07 | -1.79 | 0.40 | .0948 |
|  |  | shape - texture | -0.68 | 0.15 | -4.44 | 1.30 | .0006* |
| VO1 | frequency | shape | 0.86 | 0.18 | 4.83 | 1.08 | .0003* |
|  |  | texture | -0.06 | 0.04 | -1.55 | 0.35 | .1433 |
|  |  | shape - texture | -0.91 | 0.18 | -5.10 | 1.60 | .0002* |
|  | orientation | shape | 0.90 | 0.22 | 4.13 | 0.92 | .0010* |
|  |  | texture | -0.34 | 0.06 | -5.46 | 1.22 | .0001* |
|  |  | shape - texture | -1.24 | 0.26 | -4.80 | 1.73 | .0003* |
| VO2 | frequency | shape | 1.35 | 0.13 | 10.34 | 2.31 | <.0001* |
|  |  | texture | -0.14 | 0.03 | -5.43 | 1.21 | .0001* |
|  |  | shape - texture | -1.49 | 0.13 | -11.06 | 3.54 | <.0001* |
|  | orientation | shape | 1.28 | 0.17 | 7.55 | 1.69 | <.0001* |
|  |  | texture | -0.38 | 0.06 | -5.98 | 1.34 | <.0001* |
|  |  | shape - texture | -1.66 | 0.21 | -7.79 | 2.89 | <.0001* |
| PHC1 | frequency | shape | 1.32 | 0.19 | 6.94 | 1.55 | <.0001* |
|  |  | texture | -0.19 | 0.03 | -6.04 | 1.35 | <.0001* |
|  |  | shape - texture | -1.51 | 0.19 | -7.94 | 2.48 | <.0001* |
|  | orientation | shape | 1.36 | 0.15 | 9.00 | 2.01 | <.0001* |
|  |  | texture | -0.32 | 0.06 | -4.93 | 1.10 | .0002* |
|  |  | shape - texture | -1.68 | 0.18 | -9.46 | 3.23 | <.0001* |
| PHC2 | frequency | shape | 1.39 | 0.20 | 7.07 | 1.58 | <.0001* |
|  |  | texture | -0.13 | 0.02 | -6.64 | 1.49 | <.0001* |
|  |  | shape - texture | -1.52 | 0.21 | -7.42 | 2.43 | <.0001* |
|  | orientation | shape | 1.25 | 0.15 | 8.57 | 1.92 | <.0001* |
|  |  | texture | -0.39 | 0.08 | -4.70 | 1.05 | .0004* |
|  |  | shape - texture | -1.63 | 0.20 | -8.27 | 3.09 | <.0001* |

**Supplementary Table 2.** Results for retinotopic regions in experiment 1. p values were corrected for multiple comparisons using a Benjamini-Hochberg correction across 150 comparisons (25 ROIs, 2 filter types, 3 model contrasts). * p < .05

| Region | Filter | Category | Adaptation type | Signal change (%) | t | d_z_ | p |
| --- | --- | --- | --- | --- | --- | --- | --- |
| OFA | frequency | face | shape | 0.05 | 0.82 | 0.17 | 0.658 |
|  |  |  | texture | 0.06 | 0.79 | 0.16 | 0.658 |
|  |  |  | shape - texture | -0.01 | -0.10 | 0.02 | 0.919 |
|  |  | house | shape | 0.14 | 3.20 | 0.65 | 0.024* |
|  |  |  | texture | 0.01 | 0.20 | 0.04 | 0.919 |
|  |  |  | shape - texture | 0.13 | 1.67 | 0.34 | 0.323 |
|  | orientation | face | shape | 0.12 | 1.89 | 0.39 | 0.284 |
|  |  |  | texture | 0.11 | 1.64 | 0.34 | 0.284 |
|  |  |  | shape - texture | 0.01 | 0.16 | 0.03 | 0.876 |
|  |  | house | shape | 0.04 | 0.73 | 0.15 | 0.708 |
|  |  |  | texture | -0.04 | -0.48 | 0.10 | 0.765 |
|  |  |  | shape - texture | 0.08 | 1.52 | 0.31 | 0.284 |
| pSTS | frequency | face | shape | 0.01 | 0.18 | 0.04 | 0.858 |
|  |  |  | texture | 0.05 | 0.62 | 0.13 | 0.764 |
|  |  |  | shape - texture | -0.04 | -0.48 | 0.10 | 0.764 |
|  |  | house | shape | 0.05 | 0.73 | 0.15 | 0.764 |
|  |  |  | texture | -0.12 | -1.09 | 0.22 | 0.764 |
|  |  |  | shape - texture | 0.17 | 2.13 | 0.43 | 0.264 |
|  | orientation | face | shape | 0.09 | 2.12 | 0.43 | 0.271 |
|  |  |  | texture | 0.10 | 1.49 | 0.30 | 0.449 |
|  |  |  | shape - texture | 0.00 | -0.07 | 0.01 | 0.944 |
|  |  | house | shape | 0.03 | 0.41 | 0.08 | 0.820 |
|  |  |  | texture | -0.03 | -0.43 | 0.09 | 0.820 |
|  |  |  | shape - texture | 0.05 | 1.03 | 0.21 | 0.630 |
| OPA | frequency | face | shape | 0.01 | 0.24 | 0.05 | 0.810 |
|  |  |  | texture | 0.08 | 1.42 | 0.29 | 0.340 |
|  |  |  | shape - texture | -0.07 | -1.09 | 0.22 | 0.432 |
|  |  | house | shape | 0.17 | 2.91 | 0.59 | 0.047* |
|  |  |  | texture | 0.05 | 0.64 | 0.13 | 0.637 |
|  |  |  | shape - texture | 0.11 | 1.80 | 0.37 | 0.256 |
|  | orientation | face | shape | 0.08 | 2.28 | 0.47 | 0.092 |
|  |  |  | texture | 0.06 | 1.37 | 0.28 | 0.278 |
|  |  |  | shape - texture | 0.02 | 0.43 | 0.09 | 0.670 |
|  |  | house | shape | 0.09 | 2.11 | 0.43 | 0.092 |
|  |  |  | texture | 0.12 | 2.21 | 0.45 | 0.092 |
|  |  |  | shape - texture | -0.03 | -0.54 | 0.11 | 0.670 |
| RSC | frequency | face | shape | 0.05 | 1.23 | 0.25 | 0.578 |
|  |  |  | texture | 0.00 | 0.01 | 0.00 | 0.996 |
|  |  |  | shape - texture | 0.05 | 1.66 | 0.34 | 0.578 |
|  |  | house | shape | 0.03 | 0.89 | 0.18 | 0.578 |
|  |  |  | texture | 0.04 | 0.94 | 0.19 | 0.578 |
|  |  |  | shape - texture | -0.01 | -0.18 | 0.04 | 0.996 |
|  | orientation | face | shape | 0.00 | -0.10 | 0.02 | 0.923 |
|  |  |  | texture | 0.01 | 0.20 | 0.04 | 0.923 |
|  |  |  | shape - texture | -0.01 | -0.29 | 0.06 | 0.923 |
|  |  | house | shape | -0.02 | -0.80 | 0.16 | 0.864 |
|  |  |  | texture | -0.07 | -1.44 | 0.29 | 0.864 |
|  |  |  | shape - texture | 0.04 | 0.97 | 0.20 | 0.864 |

**Supplementary Table 3.** Results for additional face- and scene-selective regions in experiment 2. Shape adaptation is defined as shape change – no change; texture adaptation is defined as texture change – no change. p values were corrected for multiple comparisons using a Benjamini-Hochberg correction across 6 comparisons for each ROI/filter combination. * p < .05.

| Region | Filter | | Category | | Adaptation type | | Signal change (%) | | t | d_z_ | | p |  |
| --- | --- | --- | --- | --- | --- | --- | --- | --- | --- | --- | --- | --- | --- |
| V1d | frequency | | face | | shape | -0.02 | | -0.38 | | 0.08 | | 0.845 |  |
|  |  | |  | | texture | -0.03 | | -0.41 | | 0.08 | | 0.845 |  |
|  |  | |  | | shape - texture | 0.01 | | 0.12 | | 0.02 | | 0.907 |  |
|  |  | | house | | shape | 0.02 | | 0.39 | | 0.08 | | 0.845 |  |
|  |  | |  | | texture | -0.06 | | -0.86 | | 0.18 | | 0.845 |  |
|  |  | |  | | shape - texture | 0.08 | | 1.37 | | 0.28 | | 0.845 |  |
|  | orientation | | face | | shape | 0.06 | | 1.57 | | 0.32 | | 0.728 |  |
|  |  | |  | | texture | 0.02 | | 0.51 | | 0.11 | | 0.734 |  |
|  |  | |  | | shape - texture | 0.04 | | 0.88 | | 0.18 | | 0.728 |  |
|  |  | | house | | shape | 0.04 | | 0.84 | | 0.17 | | 0.728 |  |
|  |  | |  | | texture | 0.00 | | -0.09 | | 0.02 | | 0.929 |  |
|  |  | |  | | shape - texture | 0.04 | | 0.71 | | 0.14 | | 0.728 |  |
| V2d | frequency | | face | | shape | -0.05 | | -0.92 | | 0.19 | | 0.647 |  |
|  |  | |  | | texture | -0.07 | | -1.07 | | 0.22 | | 0.647 |  |
|  |  | |  | | shape - texture | 0.02 | | 0.29 | | 0.06 | | 0.776 |  |
|  |  | | house | | shape | 0.05 | | 0.64 | | 0.13 | | 0.647 |  |
|  |  | |  | | texture | -0.06 | | -0.62 | | 0.13 | | 0.647 |  |
|  |  | |  | | shape - texture | 0.10 | | 1.50 | | 0.31 | | 0.647 |  |
|  | orientation | | face | | shape | 0.07 | | 1.37 | | 0.28 | | 0.867 |  |
|  |  | |  | | texture | 0.04 | | 0.51 | | 0.10 | | 0.867 |  |
|  |  | |  | | shape - texture | 0.03 | | 0.54 | | 0.11 | | 0.867 |  |
|  |  | | house | | shape | 0.01 | | 0.15 | | 0.03 | | 0.882 |  |
|  |  | |  | | texture | -0.02 | | -0.36 | | 0.07 | | 0.867 |  |
|  |  | |  | | shape - texture | 0.03 | | 0.36 | | 0.07 | | 0.867 |  |
| V3d | frequency | | face | | shape | -0.04 | | -0.68 | | 0.14 | | 0.732 |  |
|  |  | |  | | texture | -0.02 | | -0.35 | | 0.07 | | 0.732 |  |
|  |  | |  | | shape - texture | -0.02 | | -0.35 | | 0.07 | | 0.732 |  |
|  |  | | house | | shape | 0.11 | | 1.54 | | 0.31 | | 0.411 |  |
|  |  | |  | | texture | -0.04 | | -0.37 | | 0.08 | | 0.732 |  |
|  |  | |  | | shape - texture | 0.15 | | 1.62 | | 0.33 | | 0.411 |  |
|  | orientation | | face | | shape | 0.05 | | 0.86 | | 0.18 | | 0.918 |  |
|  |  | |  | | texture | 0.04 | | 0.54 | | 0.11 | | 0.918 |  |
|  |  | |  | | shape - texture | 0.01 | | 0.10 | | 0.02 | | 0.918 |  |
|  |  | | house | | shape | 0.02 | | 0.34 | | 0.07 | | 0.918 |  |
|  |  | |  | | texture | 0.01 | | 0.13 | | 0.03 | | 0.918 |  |
|  |  | |  | | shape - texture | 0.01 | | 0.19 | | 0.04 | | 0.918 |  |
| V3a | frequency | | face | | shape | 0.02 | | 0.38 | | 0.08 | | 0.908 |  |
|  |  | |  | | texture | 0.02 | | 0.24 | | 0.05 | | 0.908 |  |
|  |  | |  | | shape - texture | 0.01 | | 0.12 | | 0.02 | | 0.908 |  |
|  |  | | house | | shape | 0.05 | | 0.83 | | 0.17 | | 0.834 |  |
|  |  | |  | | texture | -0.10 | | -0.98 | | 0.20 | | 0.834 |  |
|  |  | |  | | shape - texture | 0.15 | | 1.76 | | 0.36 | | 0.547 |  |
|  | orientation | | face | | shape | 0.05 | | 0.83 | | 0.17 | | 0.919 |  |
|  |  | |  | | texture | 0.07 | | 1.08 | | 0.22 | | 0.919 |  |
|  |  | |  | | shape - texture | -0.02 | | -0.26 | | 0.05 | | 0.919 |  |
|  |  | | house | | shape | 0.02 | | 0.27 | | 0.05 | | 0.919 |  |
|  |  | |  | | texture | 0.02 | | 0.42 | | 0.09 | | 0.919 |  |
|  |  | |  | | shape - texture | -0.01 | | -0.10 | | 0.02 | | 0.919 |  |
| V3b | frequency | | face | | shape | 0.03 | | 0.50 | | 0.10 | | 0.747 |  |
|  |  | |  | | texture | 0.03 | | 0.63 | | 0.13 | | 0.747 |  |
|  |  | |  | | shape - texture | 0.00 | | -0.03 | | 0.01 | | 0.976 |  |
|  |  | | house | | shape | 0.10 | | 1.58 | | 0.32 | | 0.382 |  |
|  |  | |  | | texture | -0.07 | | -0.75 | | 0.15 | | 0.747 |  |
|  |  | |  | | shape - texture | 0.17 | | 2.18 | | 0.44 | | 0.241 |  |
|  | orientation | | face | | shape | 0.06 | | 1.09 | | 0.22 | | 0.557 |  |
|  |  | |  | | texture | 0.07 | | 1.09 | | 0.22 | | 0.557 |  |
|  |  | |  | | shape - texture | -0.02 | | -0.33 | | 0.07 | | 0.746 |  |
|  |  | | house | | shape | 0.02 | | 0.43 | | 0.09 | | 0.746 |  |
|  |  | |  | | texture | 0.08 | | 1.38 | | 0.28 | | 0.557 |  |
|  |  | |  | | shape - texture | -0.05 | | -0.91 | | 0.19 | | 0.557 |  |
| LO1 | frequency | | face | | shape | 0.03 | | 0.47 | | 0.10 | | 0.835 |  |
|  |  | |  | | texture | 0.04 | | 0.74 | | 0.15 | | 0.835 |  |
|  |  | |  | | shape - texture | -0.02 | | -0.33 | | 0.07 | | 0.835 |  |
|  |  | | house | | shape | 0.10 | | 1.54 | | 0.31 | | 0.415 |  |
|  |  | |  | | texture | -0.02 | | -0.21 | | 0.04 | | 0.835 |  |
|  |  | |  | | shape - texture | 0.12 | | 1.72 | | 0.35 | | 0.415 |  |
|  | orientation | | face | | shape | 0.07 | | 1.71 | | 0.35 | | 0.341 |  |
|  |  | |  | | texture | 0.08 | | 1.64 | | 0.34 | | 0.341 |  |
|  |  | |  | | shape - texture | -0.02 | | -0.37 | | 0.08 | | 0.713 |  |
|  |  | | house | | shape | 0.03 | | 0.57 | | 0.12 | | 0.689 |  |
|  |  | |  | | texture | 0.08 | | 1.27 | | 0.26 | | 0.431 |  |
|  |  | |  | | shape - texture | -0.05 | | -0.95 | | 0.19 | | 0.525 |  |
| LO2 | frequency | | face | | shape | 0.08 | | 1.23 | | 0.25 | | 0.568 |  |
|  |  | |  | | texture | 0.03 | | 0.58 | | 0.12 | | 0.568 |  |
|  |  | |  | | shape - texture | 0.05 | | 0.85 | | 0.17 | | 0.568 |  |
|  |  | | house | | shape | 0.05 | | 0.68 | | 0.14 | | 0.568 |  |
|  |  | |  | | texture | -0.06 | | -0.64 | | 0.13 | | 0.568 |  |
|  |  | |  | | shape - texture | 0.11 | | 1.60 | | 0.33 | | 0.568 |  |
|  | orientation | | face | | shape | 0.04 | | 0.83 | | 0.17 | | 0.707 |  |
|  |  | |  | | texture | 0.05 | | 0.78 | | 0.16 | | 0.707 |  |
|  |  | |  | | shape - texture | -0.01 | | -0.29 | | 0.06 | | 0.926 |  |
|  |  | | house | | shape | 0.00 | | 0.04 | | 0.01 | | 0.969 |  |
|  |  | |  | | texture | 0.05 | | 0.73 | | 0.15 | | 0.707 |  |
|  |  | |  | | shape - texture | -0.04 | | -0.86 | | 0.18 | | 0.707 |  |
| hMT | frequency | | face | | shape | 0.06 | | 0.84 | | 0.17 | | 0.708 |  |
|  |  | |  | | texture | 0.03 | | 0.66 | | 0.13 | | 0.708 |  |
|  |  | |  | | shape - texture | 0.02 | | 0.38 | | 0.08 | | 0.708 |  |
|  |  | | house | | shape | 0.03 | | 0.40 | | 0.08 | | 0.708 |  |
|  |  | |  | | texture | -0.10 | | -0.85 | | 0.17 | | 0.708 |  |
|  |  | |  | | shape - texture | 0.13 | | 1.80 | | 0.37 | | 0.511 |  |
|  | orientation | | face | | shape | 0.06 | | 1.38 | | 0.28 | | 0.884 |  |
|  |  | |  | | texture | 0.05 | | 0.69 | | 0.14 | | 0.884 |  |
|  |  | |  | | shape - texture | 0.02 | | 0.34 | | 0.07 | | 0.884 |  |
|  |  | | house | | shape | 0.00 | | 0.02 | | 0.00 | | 0.988 |  |
|  |  | |  | | texture | 0.03 | | 0.50 | | 0.10 | | 0.884 |  |
|  |  | |  | | shape - texture | -0.03 | | -0.61 | | 0.12 | | 0.884 |  |
| MST | | frequency | | face | shape | 0.04 | | 1.03 | | 0.21 | 0.622 | | |
|  | |  | |  | texture | 0.00 | | 0.11 | | 0.02 | 0.939 | | |
|  | |  | |  | shape - texture | 0.04 | | 0.83 | | 0.17 | 0.622 | | |
|  | |  | | house | shape | 0.00 | | 0.08 | | 0.02 | 0.939 | | |
|  | |  | |  | texture | -0.06 | | -0.97 | | 0.20 | 0.622 | | |
|  | |  | |  | shape - texture | 0.07 | | 1.41 | | 0.29 | 0.622 | | |
|  | | orientation | | face | shape | 0.06 | | 2.03 | | 0.41 | 0.163 | | |
|  | |  | |  | texture | 0.04 | | 1.23 | | 0.25 | 0.345 | | |
|  | |  | |  | shape - texture | 0.01 | | 0.40 | | 0.08 | 0.690 | | |
|  | |  | | house | shape | 0.07 | | 2.15 | | 0.44 | 0.163 | | |
|  | |  | |  | texture | 0.05 | | 1.71 | | 0.35 | 0.201 | | |
|  | |  | |  | shape - texture | 0.03 | | 0.75 | | 0.15 | 0.553 | | |
| V1v | | frequency | | face | shape | -0.02 | | -0.36 | | 0.07 | 0.866 | | |
|  | |  | |  | texture | 0.03 | | 0.39 | | 0.08 | 0.866 | | |
|  | |  | |  | shape - texture | -0.05 | | -0.78 | | 0.16 | 0.866 | | |
|  | |  | | house | shape | 0.04 | | 0.80 | | 0.16 | 0.866 | | |
|  | |  | |  | texture | 0.00 | | -0.05 | | 0.01 | 0.960 | | |
|  | |  | |  | shape - texture | 0.04 | | 0.65 | | 0.13 | 0.866 | | |
|  | | orientation | | face | shape | 0.02 | | 0.42 | | 0.09 | 0.977 | | |
|  | |  | |  | texture | 0.02 | | 0.31 | | 0.06 | 0.977 | | |
|  | |  | |  | shape - texture | 0.00 | | 0.03 | | 0.01 | 0.977 | | |
|  | |  | | house | shape | 0.04 | | 1.05 | | 0.21 | 0.977 | | |
|  | |  | |  | texture | 0.01 | | 0.12 | | 0.02 | 0.977 | | |
|  | |  | |  | shape - texture | 0.04 | | 0.75 | | 0.15 | 0.977 | | |
| V2v | | frequency | | face | shape | 0.01 | | 0.10 | | 0.02 | 0.921 | | |
|  | |  | |  | texture | 0.03 | | 0.41 | | 0.08 | 0.921 | | |
|  | |  | |  | shape - texture | -0.02 | | -0.40 | | 0.08 | 0.921 | | |
|  | |  | | house | shape | 0.03 | | 0.71 | | 0.14 | 0.921 | | |
|  | |  | |  | texture | -0.01 | | -0.17 | | 0.03 | 0.921 | | |
|  | |  | |  | shape - texture | 0.04 | | 0.67 | | 0.14 | 0.921 | | |
|  | | orientation | | face | shape | 0.03 | | 0.83 | | 0.17 | 0.764 | | |
|  | |  | |  | texture | 0.03 | | 0.67 | | 0.14 | 0.764 | | |
|  | |  | |  | shape - texture | 0.00 | | -0.01 | | 0.00 | 0.992 | | |
|  | |  | | house | shape | 0.05 | | 1.45 | | 0.30 | 0.764 | | |
|  | |  | |  | texture | 0.01 | | 0.18 | | 0.04 | 0.992 | | |
|  | |  | |  | shape - texture | 0.05 | | 1.08 | | 0.22 | 0.764 | | |
| V3v | | frequency | | face | shape | 0.04 | | 1.25 | | 0.26 | 0.521 | | |
|  | |  | |  | texture | 0.05 | | 1.27 | | 0.26 | 0.521 | | |
|  | |  | |  | shape - texture | -0.01 | | -0.26 | | 0.05 | 0.927 | | |
|  | |  | | house | shape | 0.03 | | 1.15 | | 0.24 | 0.521 | | |
|  | |  | |  | texture | 0.00 | | 0.09 | | 0.02 | 0.927 | | |
|  | |  | |  | shape - texture | 0.03 | | 0.58 | | 0.12 | 0.852 | | |
|  | | orientation | | face | shape | 0.05 | | 1.72 | | 0.35 | 0.183 | | |
|  | |  | |  | texture | 0.05 | | 1.83 | | 0.37 | 0.183 | | |
|  | |  | |  | shape - texture | 0.00 | | -0.08 | | 0.02 | 0.934 | | |
|  | |  | | house | shape | 0.06 | | 2.22 | | 0.45 | 0.183 | | |
|  | |  | |  | texture | 0.05 | | 1.61 | | 0.33 | 0.183 | | |
|  | |  | |  | shape - texture | 0.01 | | 0.44 | | 0.09 | 0.797 | | |
| hV4 | | frequency | | face | shape | 0.06 | | 2.05 | | 0.42 | 0.105 | | |
|  | |  | |  | texture | 0.08 | | 2.54 | | 0.52 | 0.103 | | |
|  | |  | |  | shape - texture | -0.02 | | -0.84 | | 0.17 | 0.494 | | |
|  | |  | | house | shape | 0.05 | | 2.25 | | 0.46 | 0.103 | | |
|  | |  | |  | texture | 0.05 | | 1.38 | | 0.28 | 0.273 | | |
|  | |  | |  | shape - texture | 0.00 | | 0.08 | | 0.02 | 0.940 | | |
|  | | orientation | | face | shape | 0.07 | | 2.45 | | 0.50 | 0.041* | | |
|  | |  | |  | texture | 0.07 | | 3.09 | | 0.63 | 0.031* | | |
|  | |  | |  | shape - texture | -0.01 | | -0.35 | | 0.07 | 0.874 | | |
|  | |  | | house | shape | 0.07 | | 2.54 | | 0.52 | 0.041* | | |
|  | |  | |  | texture | 0.06 | | 2.36 | | 0.48 | 0.041* | | |
|  | |  | |  | shape - texture | 0.00 | | 0.10 | | 0.02 | 0.920 | | |
| VO1 | | frequency | | face | shape | 0.05 | | 1.69 | | 0.34 | 0.210 | | |
|  | |  | |  | texture | 0.09 | | 2.54 | | 0.52 | 0.109 | | |
|  | |  | |  | shape - texture | -0.03 | | -1.05 | | 0.21 | 0.455 | | |
|  | |  | | house | shape | 0.04 | | 1.92 | | 0.39 | 0.204 | | |
|  | |  | |  | texture | 0.03 | | 0.79 | | 0.16 | 0.527 | | |
|  | |  | |  | shape - texture | 0.01 | | 0.32 | | 0.06 | 0.755 | | |
|  | | orientation | | face | shape | 0.04 | | 1.54 | | 0.31 | 0.205 | | |
|  | |  | |  | texture | 0.05 | | 2.37 | | 0.48 | 0.118 | | |
|  | |  | |  | shape - texture | -0.01 | | -0.52 | | 0.11 | 0.611 | | |
|  | |  | | house | shape | 0.05 | | 1.90 | | 0.39 | 0.141 | | |
|  | |  | |  | texture | 0.06 | | 2.19 | | 0.45 | 0.118 | | |
|  | |  | |  | shape - texture | -0.01 | | -0.55 | | 0.11 | 0.611 | | |
| VO2 | | frequency | | face | shape | 0.04 | | 1.21 | | 0.25 | 0.578 | | |
|  | |  | |  | texture | 0.06 | | 1.48 | | 0.30 | 0.578 | | |
|  | |  | |  | shape - texture | -0.02 | | -0.55 | | 0.11 | 0.815 | | |
|  | |  | | house | shape | 0.03 | | 1.09 | | 0.22 | 0.578 | | |
|  | |  | |  | texture | 0.01 | | 0.17 | | 0.03 | 0.866 | | |
|  | |  | |  | shape - texture | 0.02 | | 0.42 | | 0.09 | 0.815 | | |
|  | | orientation | | face | shape | 0.02 | | 0.75 | | 0.15 | 0.863 | | |
|  | |  | |  | texture | 0.01 | | 0.51 | | 0.10 | 0.863 | | |
|  | |  | |  | shape - texture | 0.00 | | 0.17 | | 0.04 | 0.863 | | |
|  | |  | | house | shape | 0.05 | | 1.90 | | 0.39 | 0.242 | | |
|  | |  | |  | texture | 0.06 | | 1.83 | | 0.37 | 0.242 | | |
|  | |  | |  | shape - texture | -0.01 | | -0.31 | | 0.06 | 0.863 | | |
| PHC1 | | frequency | | face | shape | 0.01 | | 0.55 | | 0.11 | 0.802 | | |
|  | |  | |  | texture | 0.02 | | 0.55 | | 0.11 | 0.802 | | |
|  | |  | |  | shape - texture | 0.00 | | -0.11 | | 0.02 | 0.916 | | |
|  | |  | | house | shape | 0.01 | | 0.46 | | 0.09 | 0.802 | | |
|  | |  | |  | texture | -0.02 | | -0.43 | | 0.09 | 0.802 | | |
|  | |  | |  | shape - texture | 0.04 | | 0.64 | | 0.13 | 0.802 | | |
|  | | orientation | | face | shape | 0.01 | | 0.65 | | 0.13 | 0.626 | | |
|  | |  | |  | texture | -0.02 | | -0.77 | | 0.16 | 0.626 | | |
|  | |  | |  | shape - texture | 0.03 | | 1.35 | | 0.28 | 0.381 | | |
|  | |  | | house | shape | 0.05 | | 2.17 | | 0.44 | 0.246 | | |
|  | |  | |  | texture | 0.04 | | 1.59 | | 0.32 | 0.375 | | |
|  | |  | |  | shape - texture | 0.01 | | 0.35 | | 0.07 | 0.730 | | |
| PHC2 | | frequency | | face | shape | -0.01 | | -0.39 | | 0.08 | 0.932 | | |
|  | |  | |  | texture | -0.01 | | -0.42 | | 0.09 | 0.932 | | |
|  | |  | |  | shape - texture | 0.02 | | -0.08 | | 0.02 | 0.932 | | |
|  | |  | | house | shape | -0.01 | | -0.21 | | 0.04 | 0.932 | | |
|  | |  | |  | texture | -0.08 | | -1.28 | | 0.26 | 0.874 | | |
|  | |  | |  | shape - texture | 0.07 | | 1.08 | | 0.22 | 0.874 | | |
|  | | orientation | | face | shape | 0.04 | | 2.08 | | 0.42 | 0.109 | | |
|  | |  | |  | texture | -0.02 | | -0.75 | | 0.15 | 0.459 | | |
|  | |  | |  | shape - texture | 0.06 | | 2.23 | | 0.46 | 0.109 | | |
|  | |  | | house | shape | 0.04 | | 2.03 | | 0.41 | 0.109 | | |
|  | |  | |  | texture | 0.02 | | 0.82 | | 0.17 | 0.459 | | |
|  | |  | |  | shape - texture | 0.03 | | 1.13 | | 0.23 | 0.407 | | |

**Supplementary Table 4.** Results for retinotopic regions in experiment 2. p values were corrected for multiple comparisons using a Benjamini-Hochberg correction across 6 comparisons in each ROI/filter combination. * p < .05.


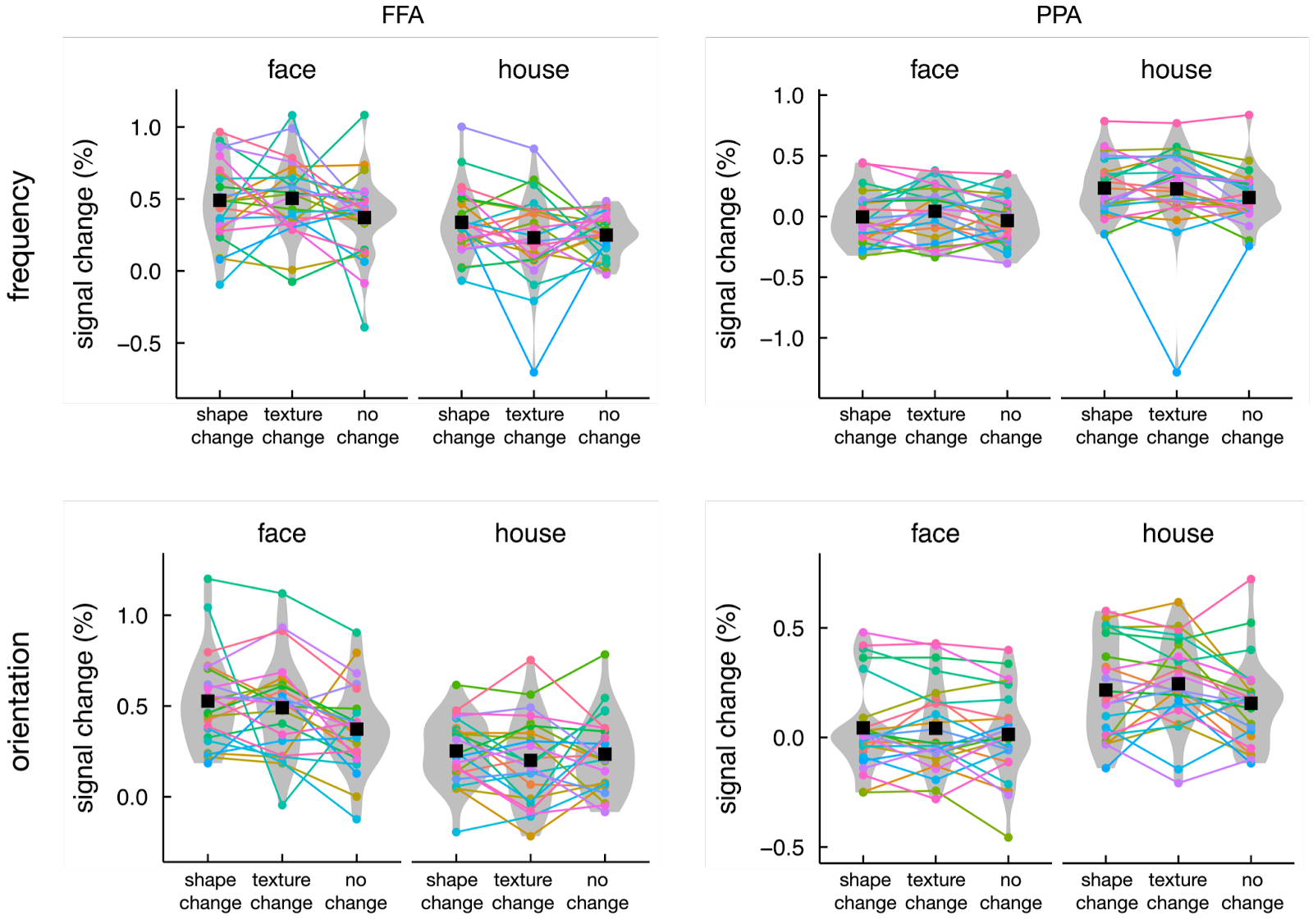


**Supplementary Figure 1.**   Response in FFA and PPA to different conditions from experiment 2. Data were separated across frequency and orientation filters.
